# 933. Incidence and Risk Factors for *Cytomegalovirus* Infection in Intestinal Transplant Recipients

**DOI:** 10.1093/ofid/ofab466.1128

**Published:** 2021-12-04

**Authors:** Anmary A Fernandez, Jacques Simkins, Eric Martin, Shweta Anjan, Jennifer Garcia, Christopher O’Brien, Rodrigo Vianna, Yoichiro Natori

**Affiliations:** 1 Jackson Memorial Hospital/Miami Transplant Institute; University of Miami School of Medicine, Tampa, FL; 2 University of Miami/Jackson Memorial Hospital, Miami, FL; 3 Jackson Memorial Hospital/Miami Transplant Institute, Miami, FL; 4 Jackson Memorial Hospital/Miami Transplant Institute, University of Miami Miller School of Medicine, Miami, FL

## Abstract

**Background:**

Cytomegalovirus (CMV) infection is the most common infection after solid organ transplantation. Data on CMV infection in intestinal transplant recipients is limited.

**Methods:**

This is a single-center retrospective cohort study which includes all consecutive intestinal transplant recipients who were transplanted between 2009 and 2019. We excluded recipients that showed CMV seronegativity of both donor and recipient. We also excluded those patients who did not have more than 3 months of follow-up. Univariate and multivariate analyses were performed to identify the risk factors for CMV infection. Of note, at our center CMV prophylaxis in intestinal transplant recipients is one year of valganciclovir.

**Results:**

A total of 173 recipients were transplanted; 46 recipients were because of CMV serostatus and 32 due to short follow-up. Ninety-five recipients were included finally. The characteristics of our cohort are summarized in Table 1. Of note, the median age was 32 years [range 0-67] and 44 (46.3%) were male. Eighteen (18.9%) recipients needed to stop valganciclovir prophylaxis due to the side effect, especially cytopenia. Twenty-one recipients developed CMV infection including asymptomatic viremia (12/21, 57.1%), CMV syndrome (5/21, 23.8%) and end-organ disease (2 (9.5%) pneumonitis and 2 (9.5%) colitis) at median time of 155 [Interquartile range, IQR 28-254] days from transplant. The median peak viral load and time to negativity were 16000 [IQR 1500-43892] IU/ml and 56 [IQR 49-109] days, respectively. Younger age (p=0.007, Odds ratio 1.03, 95% confidence interval 1.003-1.055) was the independent factor associated with CMV infection.

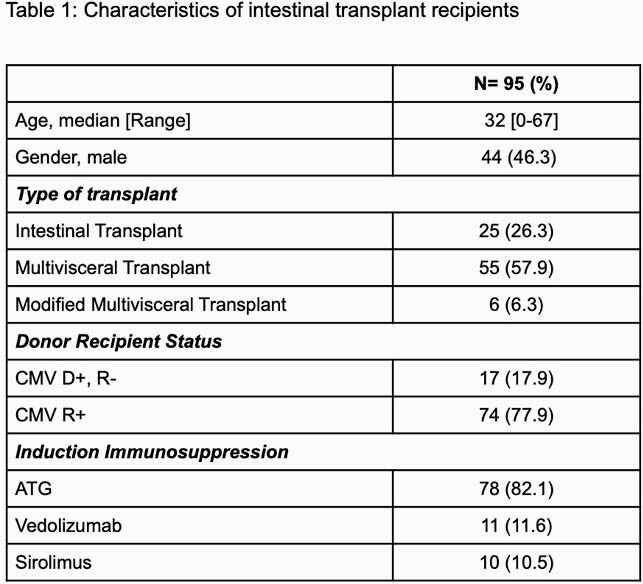

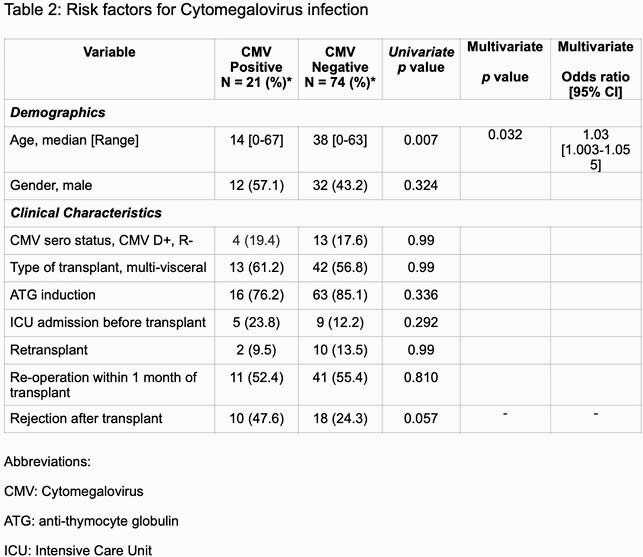

**Conclusion:**

Despite prolonged prophylaxis, 21 (22.1%) of intestinal transplant recipients developed CMV infection around 5 months post-transplant. This may be because they cannot tolerate valganciclovir prophylaxis and early termination was required. Further strategy should be developed to prevent CMV infection in this vulnerable population.

**Disclosures:**

**All Authors**: No reported disclosures

